# A protocol for a systematic review of the psychosocial effects of experiencing compassion from others and its interpretation through secular and religious frameworks

**DOI:** 10.1186/s13643-026-03093-1

**Published:** 2026-02-24

**Authors:** Carlo Lazzari, Paul Crawford, Alison Ashmore, Yasuhiro Kotera

**Affiliations:** 1 School of Health Sciences, Medical School: Queen’s Medical Centre, University of Nottingham, B236, Nottingham, NG7 2HA UK; 2 Libraries Research Support, University of Nottingham, Nottingham, UK; 3 Center for Infectious Disease Education and Research, The University of Osaka, Osaka, Japan; 4 Department of Social Sciences, Azerbaijan University, Baku, Azerbaijan

**Keywords:** Compassion, Compassion from others, Systematic review, Protocol, Mental health, Prosocial behaviors, Religion, Secularism

## Abstract

**Background:**

Compassion is widely studied as a prosocial motivator with recognized mental health benefits, yet the psychosocial effects of receiving compassion remain underexplored. Interpretive frameworks—whether religious or secular—may influence how individuals perceive and emotionally respond to compassionate acts.

**Objectives:**

To systematically review (1) the psychosocial effects of receiving compassion from others, and (2) how recipients interpret these experiences through secular or religious frameworks.

**Methods:**

This protocol follows the Preferred Reporting Items for Systematic Review and Meta-Analysis Protocols (PRISMA-P) guidelines and is registered with the International Prospective Register of Systematic Reviews (PROSPERO). Eligible studies will be empirical, involving adult participants in any setting, and using qualitative, quantitative, or mixed-method designs. Studies will be excluded if they focus solely on self-compassion, compassion fatigue, or lack psychological outcome data related to receiving compassion. Information sources include PubMed, Psychological Information Database (PsycINFO), Web of Science, Scopus, and Google Scholar. Risk of bias will be assessed using the Risk Of Bias In Non-randomized Studies of Exposures (ROBINS-E) tool for nonrandomized studies, the Joanna Briggs Institute (JBI) checklist for quasi-experimental and qualitative designs, and the Mixed Methods Appraisal Tool (MMAT) for mixed-methods designs. Data synthesis will follow a convergent integrated approach combining thematic and narrative techniques. Where feasible, pooled effect sizes and forest plots will be presented.

**Discussion:**

This review will explore how receiving compassion—interpreted through secular or religious lenses—affects psychological well-being and social interactions, including potential reductions in distress, increases in resilience, and prosocial behaviors. It aims to develop culturally sensitive and spiritually aware models of compassionate interaction, with relevance for clinical practice, public health ethics, and interdisciplinary education.

**Systematic review registration:**

PROSPERO 2025 CRD420251107986. Available from: https://www.crd.york.ac.uk/PROSPERO/view/CRD420251107986.

## Background

Compassion involves recognizing others’ suffering and responding with kindness, empathy, and a desire to help [1], which strengthens social connections and mental well-being [2]. It manifests through supportive gestures, altruistic actions, and kind communication [3] and plays a key role in fostering resilience [4]. Receiving compassion is a dynamic interpersonal process characterized by warmth, emotional presence, and attuned responses during times of distress [5]. Such care may come from loved ones, professionals, or people interacted with occasionally, influencing emotional regulation, psychological resilience, and attachment security [6]. Cultural, philosophical, and spiritual beliefs shape how compassion is received and understood [7]. From a secular perspective, it is considered a prosocial instinct rooted in evolutionary and moral theories [8], reinforced through interventions such as mindfulness and cognitive-behavioral programs [9]. Conversely, religious traditions often view compassion as a divine attribute or sacred duty [10], with rituals and teachings elevating its spiritual significance [11]. Experiencing compassion enhances psychological well-being by providing emotional safety and fostering a sense of belonging [12]. It activates calming physiological responses [13], facilitates emotion regulation, and builds trust; it is also associated with increased self-compassion, which reduces self-criticism and promotes healthier coping strategies [14]. In therapeutic settings, compassionate relationships foster healing through safety and openness [15], whereas in daily life, compassion improves mood, life satisfaction, and relational bonds [16].

Compassion is generally understood as a quality of being sensitive to the suffering of oneself or others, combined with a dedication to reduce and prevent that suffering [17]. It is not merely an emotional reaction but a complex construct involving cognitive awareness, emotional resonance, motivational intent, and behavioral action. Unlike empathy, which involves feeling with someone, compassion includes a proactive desire to help [18]. In psychosocial terms, compassion is linked to greater well-being, prosocial behavior, and resilience and is regarded as a skill that can be developed within therapeutic and contemplative settings [17, 19, 20]. Compassion from others means receiving caring attention, support, or acts of kindness. It involves recognizing that someone else has seen one’s distress and wants to help through genuine actions, support, or protection. This type of compassion is relational and often relies on trust, perceived sincerity, and cultural norms [21]. Receiving compassion activates brain neural systems associated with safety and connection, thereby helping to reduce shame, isolation, and psychological distress [22, 23]. It involves perceived vulnerability and the ability to accept care, which are shaped by individual differences such as attachment styles or fear of compassion, as well as sociocultural influences that determine whether help is perceived as empowering or stigmatizing [24]. Resistance to accepting compassion from others may stem from internalized beliefs about self-worth or concerns related to reciprocity, dependence, or autonomy [25]. Interpretations of compassion vary across religious and secular frameworks. Religiously, compassion is often considered a divine attribute or an ethical obligation, for instance, reflecting divine love in Christianity or cultivating karuṇā in Buddhism to alleviate suffering [26]. These views embed compassion within spiritual narratives of salvation and moral duty. Secular perspectives approach compassion through humanistic and evolutionary lenses, viewing it as an adaptive caregiving instinct or civic virtue that supports societal cohesion [9, 27]. Secular ethics promote compassion based on reason, empathy, and shared humanity, independent of religious belief systems [28]. Why is it essential to study compassion? Compassion significantly contributes to improved mental health by reducing stress, increasing emotional resilience, and enhancing overall life satisfaction [29–32]. It functions as a psychological asset that helps regulate emotions and alleviate distress [29–32]. In addition, compassion influences ethical decision-making and social behavior [29–32]. Its expression and understanding are shaped by moral frameworks, both secular and religious, that guide interpersonal actions and responsibilities [7, 33, 34]. In this way, compassion operates as both an emotional and an ethical force. Cultural and philosophical traditions further define how compassion is interpreted and valued. Religious teachings, societal norms, and philosophical views all play roles in shaping its expression [35, 36]. This diversity calls for culturally responsive models in compassion research that emphasize the broad relevance of compassion to mental health, moral development, prosocial behaviors, and cross-cultural understanding. What are the limitations of existing compassion research? Several issues constrain current research on receiving compassion. Most studies focus on Western contexts, which limits their cultural applicability [37–39]. Furthermore, measures often rely on inconsistent self-report tools, thereby reducing comparability across studies [24]. The use of cross-sectional designs hinders the understanding of long-term effects [23, 40]. Conceptually, definitions of compassion vary widely, leading to confusion and inconsistent measurement [8, 22]. Additionally, marginalized populations are underrepresented, thus overlooking how structural disadvantage influences responses to compassion [41]. Future work should incorporate cultural diversity, provide more precise definitions, employ longitudinal methods, and promote broader inclusion [42].

Compassion is widely recognized as a prosocial orientation involving sensitivity to suffering and a commitment to alleviate it through kindness, empathy, and supportive action [8, 43]. It manifests externally through gestures of care, altruistic behavior, and emotionally attuned communication, contributing to social cohesion and psychological resilience [22, 44]. In clinical and psychological contexts, compassion is associated with improved mental health, reduced distress, and enhanced emotion regulation [12, 45]. A critical distinction must be made between *compassion from others* and *receiving compassion*. Compassion from others refers to the observable expression of care—acts of kindness, protective support, or empathic presence offered by another person [3, 46]. It is relational and externally enacted, shaped by cultural norms, perceived sincerity, and interpersonal trust [3, 46]. This form of compassion may be delivered by loved ones, professionals, or strangers, and is typically assessed through behavioral indicators or third-party reports. Receiving compassion, by contrast, denotes the internal, subjective experience of being cared for [47, 48]. It involves perceiving and emotionally integrating another’s compassionate actions, characterized by feelings of warmth, safety, and connection during times of distress [47, 48]. This process activates brain systems associated with social bonding and threat reduction [47, 48], and contributes to psychological resilience, emotion regulation, and attachment security [49, 50].

Receiving compassion is not a given. Even when compassion is extended, individuals may be unable to feel it because of internalized beliefs about their own value, fears of relying on others, or cultural stigma associated with seeking support [51, 52]. Personal characteristics—including attachment patterns, traumatic experiences, and fears related to compassion—shape whether care is interpreted as supportive or as potentially threatening. Sociocultural expectations further influence how receiving compassion is understood and judged, particularly in environments that prize autonomy over vulnerability [53, 54].

Interpretive lenses—religious or secular—also shape how compassion is defined and taken in. Within religious traditions, compassion is often regarded as a divine quality or moral duty, embedded in narratives of salvation, obligation, and sacred connection [55, 56]. For instance, Christianity presents compassion as an expression of divine love, whereas Buddhism cultivates *karuṇā* as a disciplined practice aimed at relieving suffering [57, 58]. Secular accounts, by contrast, tend to frame compassion as an evolved caregiving instinct or a civic virtue grounded in empathy, rationality, and shared human experience [59, 60].

Experiencing compassion—whether religiously or secularly framed—enhances psychological well-being by fostering emotional safety, reducing shame, and promoting relational trust [61, 62]. In therapeutic settings, compassionate relationships facilitate healing through attunement and acceptance [2, 63]. In everyday life, receiving compassion improves mood, life satisfaction, and social connectedness [44, 64].

How current is this evidence? Compassion research has grown substantially over the past decade, with more contributions from psychology, neuroscience, theology, and public health [65]. Recent studies examine compassion as a psychological process, a neurobiological mechanism, and a culturally rooted virtue [66]. International work from South Asia and southern Africa has introduced wider cultural models [67], while clinical research has examined compassion-focused meditation and its impact on resilience and neural plasticity [68]. Religious traditions, such as Christianity, Buddhism, and Islam, continue to influence spiritually integrated interventions, framing compassion as a moral or sacred duty [26, 27]. Nonetheless, the variability in measurement tools, limited long-term studies, and lack of integration between secular and theological models remain ongoing challenges [69–71]. A systematic review is needed to unify this multidisciplinary evidence and inform future research and practice.

### Aims

Our systematic review will address two related objectives using the PI/ECOS framework. First, it will examine the psychological effects of receiving compassion (Intervention/Exposure) among adult populations in communities, organizations, and clinical settings (Population), focusing on outcomes such as psychological well-being, emotional regulation, mental health symptoms, interpersonal trust, and prosocial behaviors (Outcomes). The studies included in the review will compare receiving versus not receiving compassion, fear versus acceptance of compassion, and secular versus religious frameworks for interpreting compassion (Comparator) and adopt qualitative, quantitative, and mixed-methods research designs (Study Design) in organizations or social life (Settings). Second, the review will explore how individuals interpret acts of compassion from others, specifically through religious or secular lenses (subgroup analysis in Exposure) (Table 1). It will assess whether these interpretive frameworks influence the psychological significance or subjective impact of compassionate encounters. By integrating the psychological and interpretive aspects of compassion, the review aims to inform culturally and spiritually sensitive interventions and support more contextually grounded understandings of the role of compassion in human well-being (Table 1).

**Table 1:** PICOS inclusion and exclusion criteria

Component	Description	Inclusion criteria	Exclusion criteria
Population	Adults in communities or organizations	Adults (18 +) in clinical and nonclinical, community, and organizational settings. Therefore, adult population either in community or inpatient settings in hospital, including patients or health carers, will be included	Children, adolescents (age < 18 years), or clinical inpatient populations. In case of mixed studies with adult and children population, the study will be excluded due to statistical heterogeneity
Intervention/exposure	Receiving compassion from others, interpreted through secular or religious frameworks	Acts of compassion received; interpretation via secular or religious lens	Self-compassion studies, compassion fatigue; interventions without an interpersonal compassion component
Comparator	Not receiving compassion; fear of compassion; different interpretative frameworks	Control or comparison groups with no compassion exposure; fear vs. acceptance; receiving vs. not receiving	Studies without comparators; unrelated psychological constructs
Outcomes	Psychological well-being, emotional regulation, mental health symptoms, interpersonal trust	Outcomes related to mental and emotional states or interpersonal trust	Physical health outcomes unrelated to psychology
Study design	Qualitative, quantitative, mixed-methods	Empirical studies using qualitative, quantitative, or mixed methodologies; other systematic reviews in the case of an umbrella study	Theoretical papers, gray literature, opinion pieces, or editorials
Subgroup analysis	Interpretation of compassion through secular vs. religious lenses	Data on individuals’ perception and meaning-making of compassion via cultural/religious contexts	No information on interpretive framework; purely behavioral data

## Methods

### Review question

Our PI/ECO question is framed as follows: “How does receiving compassion from others affect psychological well-being and prosocial behaviors in communities and organizations? How does that effect map onto the secular and religious compassion frameworks?”.

### Rationale

Compassion is widely recognized as a prosocial motivator with established benefits for mental health, including reductions in distress and improvements in resilience. However, the majority of existing research has focused on self-compassion, compassion fatigue, or the act of giving compassion, leaving the psychological effects of receiving compassion from others comparatively underexplored. Furthermore, interpretive frameworks—whether religious, secular, or culturally embedded—may shape how individuals perceive and emotionally respond to compassionate acts. Understanding these dynamics is essential for developing context-sensitive models of care and for informing interventions that are ethically and culturally attuned. This review addresses a critical gap by synthesizing evidence on how receiving compassion influences psychological outcomes, prosocial behaviors, and how these experiences are interpreted across diverse belief systems and settings.

#### Objectives

This systematic review will address the following questions using a PICO framework:

- Participants (P): Adults (18 +) in any setting, including secular, community, religious, hospital, clinical, or occupational contexts.
- Intervention/Exposure (I): Receiving compassion from others—defined as intentional acts of kindness, care, or support directed toward the participant; accepting or fearing compassion from others.
- Comparator (C): Studies may include comparisons with individuals not receiving compassion, receiving neutral or negative interactions, or no comparator (in qualitative designs). Other comparators will be receiving versus not receiving compassion and accepting versus fear of receiving compassion.
- Outcomes (O): Psychological outcomes including well-being, resilience, emotional regulation, distress reduction, and interpretive responses (e.g., acceptance, fear, spiritual meaning-making). Other outcomes are prosocial behaviors such as increased empathy, greater willingness to support others, enhanced cooperative behavior, strengthened social bonding, and a heightened sense of communal responsibility. Receiving compassion can also foster gratitude, altruistic motivation, and reciprocal helping tendencies, contributing to broader patterns of social cohesion and collective care.

The review aims to (1) evaluate the psychosocial effects of receiving compassion from others and (2) explore how recipients interpret these experiences through secular or religious frameworks.

This systematic review aims to achieve the following objectives:

1. Evaluate the psychosocial effects of receiving compassion from others, including outcomes such as stress, emotional regulation, resilience, and psychological distress, among adults in community and organizational settings.
2. Examine how secular versus religious frameworks influence the interpretation of receiving compassion from others.
3. Explore the psychosocial impact of the received compassion within relational environments such as organizations, hospitals, clinical settings, and communities.
4. Compare psychological well-being between adults who report receiving compassion and those who do not, using validated psychometric measures across diverse relational contexts.

### Use of the PRISMA framework in the systematic review

This systematic review will follow the PRISMA 2020 guidelines to ensure transparency, methodological rigor, and reproducibility throughout its design and implementation [72]. Our protocol is registered on PROSPERO to define objectives, eligibility criteria, and analytical methods, thereby reducing bias and improving consistency [73]. We will adopt a comprehensive search strategy, developed in collaboration with an information specialist, which will utilize subject headings and free-text terms across multiple databases, with screening carried out independently by two reviewers. The selection results will be visualized with a PRISMA flow diagram, and the reference lists of the included studies will be reviewed for additional sources. Data extraction will use standardized, pilot-tested forms, and synthesis will account for the diversity and heterogeneity of study designs. The risk of bias will be assessed using appropriate tools for each study type, including the JBI tool for qualitative studies and the ROBINS-E tool for observational research [74]. The results will be reported in accordance with all 27 PRISMA checklist items, supported by visual tools such as flow diagrams, forest plots, and summary tables. Any deviations from the protocol will be documented, and efforts will be made to include the review in open-access repositories to support transparency and reproducibility [73]. By adopting this approach, the review aims to establish a robust foundation for evidence synthesis, yielding findings that are trustworthy and readily accessible to researchers, policymakers, and practitioners.

### Reviewing the quality of our SR

This systematic review will use the PI/ECOS (Population, Intervention or Exposure, Comparison, Outcomes, and Settings) framework and will be registered in a published protocol to ensure transparency and reproducibility [75–77]. The inclusion criteria will be appropriately defined to support relevance and allow for meta-analyses if necessary [78]. A comprehensive and justified search strategy will combine controlled vocabulary and free-text terms across core and discipline-specific databases, considering any limitations [79, 80]. Methodological quality will be assessed using the Joanna Briggs Institute (JBI) tool, which is tailored to different study designs and applied independently by two reviewers. Discrepancies will be resolved by consensus or arbitration [81–83]. Interrater reliability will be calculated for 10% of the sample using Cohen’s kappa [84, 85]. The review will address two main issues: the psychological effects of receiving compassion in relational contexts, such as schools, workplaces, and faith-based groups, and the interpretive frameworks—both religious and secular—through which compassion is understood [86–90]. Mixed-methods synthesis will combine statistical trends and narrative insights to inform culturally and spiritually responsive interventions [4, 91, 92].

### PRISMA framework

The systematic review will follow the Preferred Reporting Items for Systematic Reviews and Meta-Analysis (PRISMA) guidelines (see the Appendix). The current protocol will follow the Preferred Reporting Items for Systematic Reviews and Meta-Analysis Protocol (PRISMA-P) guidelines.

### PI/ECO framework

#### Population

The review will focus on adults aged 18 and over in community, clinical, and organizational settings where compassion may be present or absent. Sociodemographic factors, including age, gender, ethnicity, religion, and location, will be systematically extracted (Table 2). A health equity perspective, guided by PRISMA-E 2012 [4], will inform data disaggregation by socioeconomic status, urban–rural context, and cultural background, with explicit attention to marginalized groups. For qualitative evidence, the GRADE-CERQual framework [93] will be used to assess confidence across four domains: methodological limitations, coherence, data sufficiency, and relevance. Summary of Qualitative Findings tables [94] will be prepared to highlight population-specific insights and CERQual ratings, incorporating contextual quotes to shed light on lived experiences. This approach fosters transparency and provides a rich understanding of how compassion is received and interpreted among diverse adult populations [3, 95, 96].

**Table 2:** General search keywords as extracted from the SR questions

Framework	Search keywords
General PICO search	
Compassion	((empathy OR compassion* OR “compassion from other*” OR “receiving compassion*” OR “giving compassion*” OR “compassionate response*” OR “compassionate action*” OR “compassion-based intervention*” OR “compassion motivat*” OR “compassion satisfact*” OR “receiv* compassion” OR “interpersonal compassion” OR “compassionate behavio?r” OR “compassion-based support” OR “compassionate interaction*” OR “compassionate respons*” OR “compassion* receiv*”) NOT (“compassionate care” OR “self-compassion*” OR “compassion fatigue”))
Psychological well-being	(‘psychological well?being’ OR ‘mental well?being’ OR ‘emotional well?being’ OR ‘psychological distress’ OR flourish* OR ‘positive psycholog*’ OR resilien* OR ‘subjective well?being’ OR ‘mental health’)
Community or organizational contexts	(communities OR communit* OR ‘community setting*’ OR organization* OR organization* OR workplace* OR ‘work environment*’ OR ‘team dynamic*’ OR collectiv* OR ‘social group*’ OR institution*)
Secular or religious compassion frameworks	(‘secular’ OR ‘religious’ OR ‘secular compassion’ OR ‘religious compassion’ OR ‘spiritual compassion’ OR ‘theological compassion’ OR ‘faith-based’ OR ‘spiritual framework*’ OR ‘moral philosoph*’ OR ‘ethical framework*’ OR buddhis* OR agape OR dharma OR karuṇā OR Christian)
Search keywords combined adapted to MeSH database (tiab = title and abstract)	
Compassion	(“Empathy”[MeSH Terms] OR compassion*[tiab] OR “compassionate behavio?r”[tiab] OR “interpersonal compassion”[tiab] OR “compassion from other*”[tiab] OR “compassionate interaction*”[tiab] OR “compassionate respons*”[tiab] OR “compassion* receiv*”[tiab]) AND (“Helping Behavior”[MeSH Terms] OR “Altruism”[MeSH Terms] OR “Professional-Patient Relations”[MeSH Terms] OR “Interpersonal Relations”[MeSH Terms] OR “Social Support”[MeSH Terms] OR “Attitude of Health Personnel”[MeSH Terms]) AND (“compassion* receiv*”[tiab] OR “receiving compassion”[tiab] OR “perceived compassion”[tiab] OR “felt compassion”[tiab])AND
Psychological well-being	(‘Psychological Well-Being’[Mesh] OR ‘Mental Health’[Mesh] OR ‘Emotions’[Mesh] OR ‘psychological well?being’[tiab] OR ‘emotional well?being’[tiab] OR ‘mental well?being’[tiab] OR resilien*[tiab] OR flourish*[tiab] OR ‘positive psycholog*’[tiab] OR ‘psychological distress’[tiab]) AND
Community or organizational contexts	(‘Community-Institutional Relations’[Mesh] OR ‘Community Networks’[Mesh] OR ‘Organizations’[Mesh] OR ‘Workplace’[Mesh] OR communit*[tiab] OR organization*[tiab] OR organization*[tiab] OR workplace*[tiab] OR institution*[tiab] OR ‘team dynamic*’[tiab] OR collectiv*[tiab]) AND
Secular or religious compassion frameworks	(‘Religion and Psychology’[Mesh] OR ‘Spirituality’[Mesh] OR ‘Ethics’[Mesh] OR ‘Religion’[Mesh] OR ‘spiritual compassion’[tiab] OR ‘religious compassion’[tiab] OR ‘theological compassion’[tiab] OR ‘faith-based’[tiab] OR ‘secular compassion’[tiab] OR ‘moral philosoph*’[tiab] OR agape[tiab] OR karuṇā[tiab] OR dharma[tiab] OR christian*[tiab] OR buddhis*[tiab])
Search strategy tailored for APA PsycINFO, using APA Thesaurus of Psychological Index Terms (where available) alongside relevant keywords (DE = Descriptor)	
Compassion	(DE “Compassion” OR “compassion from other*” OR “interpersonal compassion” OR “compassionate behavio?r” OR “compassionate interaction*” OR “compassion* receiv*” OR “compassion-based support” OR “compassionate respons*”) AND
Psychological well-being	(DE “Psychological Well Being” OR “psychological well?being” OR “mental well?being” OR “emotional well?being” OR “psychological distress” OR flourish* OR “positive psycholog*” OR resilien* OR “subjective well?being” OR “mental health”) AND
Community or organizational contexts	(DE “Community Life” OR DE “Work Environment” OR DE “Organizational Climate” OR DE “Workplace Environment” OR communit* OR organization* OR organization* OR workplace* OR institution* OR “team dynamic*” OR collectiv* OR “social group*”) AND
Secular or religious compassion frameworks	(DE “Spirituality” OR DE “Religious Beliefs” OR DE “Religious Factors” OR “religious compassion” OR “spiritual compassion” OR “theological compassion” OR “faith-based” OR “secular compassion” OR buddhis* OR christian* OR agape OR dharma OR karuṇā OR “ethical framework*” OR “moral philosoph*”)
Web of Science search strategy using NEAR/x proximity operators for more nuanced retrieval, where x is the maximum number of words allowed between terms [TS = topic]	
Compassion	TS = (compassion* NEAR/3 (receiv* OR “from other*” OR interpersonal OR respons* OR support OR interaction* OR behavio?r)) AND
Psychological well-being	TS = (“psychological” OR “mental” OR “emotional” OR “subjective”) NEAR/3 (well?being OR health OR distress) OR flourish* OR resilien* OR “positive psycholog*”) AND
Community or organizational contexts	TS = (communit* OR “community setting*” OR organization* OR organization* OR workplace* OR institution* OR (“work” NEAR/3 environment*) OR (“team” NEAR/3 dynamic*) OR collectiv* OR “social group*”) AND
Secular or religious compassion frameworks	TS = ((“religious” OR “spiritual” OR “secular” OR “theological”) NEAR/3 compassion OR “faith-based” OR “moral philosoph*” OR “ethical framework*” OR buddhis* OR christian* OR agape OR dharma OR karuṇā)
OVID search	
1	exp Empathy/— Exploded MeSH term for empathy
2	(compassion* or empath*).mp. — Keyword search using truncation
3	1 or 2 — Combines empathy MeSH and keyword terms
4	exp Psychological Well-Being/— Exploded MeSH term
5	exp Mental Health/— Exploded MeSH term
6	exp Emotions/— Exploded MeSH term
7	(“psychological well?being” or “emotional well?being” or “mental well?being” or resilien* or flourish* or “positive psycholog*” or “psychological distress” or emotion*).mp. — Keyword search for mental and emotional health
8	4 or 5 or 6 or 7 — Combines mental/emotional well-being terms
9	exp Community-Institutional Relations/— Exploded MeSH term
10	exp Community Networks/— Exploded MeSH term
11	exp Organizations/— Exploded MeSH term
12	exp Workplace/— Exploded MeSH term
13	(communit* or workplace* or organi?ation* or institution* or “team dynamic*” or collectiv*).mp. — Keyword search for social/organizational context
14	9 or 10 or 11 or 12 or 13 — Combines community and institutional terms
15	exp Religion/— Exploded MeSH term
16	exp Spirituality/— Exploded MeSH term
17	exp Ethics/— Exploded MeSH term
18	(“spiritual compassion” or “religious compassion” or “theological compassion” or “faith-based” or “secular compassion” or “moral philosoph*” or agape or karuna or dharma or christian* or buddhis* or religion or faith or ethic* or islam*).mp. — Keyword search for religious and ethical framing
19	15 or 16 or 17 or 18 — Combines religious/spiritual/ethical terms
20	3 and 8 and 14 and 19 — Final combined logic: empathy AND well-being AND social context AND spiritual/ethical framing

#### Exposure

This review defines receiving compassion as sincere care and empathetic support during vulnerability, which are expressed through verbal, behavioral, or relational acts [97]. A mixed-methods approach will be used to explore its multidimensional nature. Quantitative data will be collected from validated self-report tools, including the Compassionate and Engagement Scales, Social Communication Questionnaire, and the Receiving from Others subscale of the Fears of Compassion Scale [98–100]. These tools will be used to analyze variables such as intensity, frequency, and source attribution, with subgroup comparisons across secular and religious contexts. Qualitative data, including interviews, focus groups, and ethnographies, will be analyzed using meta-ethnography for theory extraction [50, 101, 102]. Emotional presence, kindness, and cultural meanings of compassion will be examined to combine statistical metrics and experiential insights across diverse populations.

#### Comparators

The systematic review will identify comparators, or alternative conditions, used to evaluate the psychosocial impact of receiving compassion, thereby filling a significant gap in existing research [103]. It will examine three main comparative aspects: psychological differences between those who receive compassion and those who do not; interpretive frameworks—religious versus secular—that influence perception and response; and individual receptivity, which is affected by factors such as trauma and attachment insecurity [103]. Quantitative analysis will involve classifying participants by worldview, receptivity (e.g., Fears of Compassion Scale), and exposure level and analyzing outcomes such as emotional regulation and distress. Correlation, moderation, and covariance analyses will contribute to a comprehensive understanding that promotes ethically and culturally sensitive applications across clinical, organizational, and community contexts (Table 3).

**Table 3:** Summative comparator table for data extraction

Comparator domain	Group 1	Group 2	Measurement type	Data to extract
Interpretive framework	Religious interpretation of compassion	Secular (or nonreligious) interpretation	Nominal categorization (e.g., self-identified)	Frequency and psychological scores by interpretive category
Receptivity to compassion	Acceptance of compassion	Fear/resistance to compassion	Interval scales (e.g., fears of compassion, anxiety, depression, stress)	Mean, SD, and psychometric differences across groups
Compassion exposure	Regular recipients of compassion	Minimal or no reported compassion exposure	Interval compassion and well-being scales	Group means, SDs, and effect sizes for psychological outcomes
Psychometric correlates	Varies by subgroup	–	Bivariate analyses (e.g., Pearson’s *r*)	Correlation values between compassion and well-being scales

In qualitative studies, comparators arise from thematic and experiential contrasts rather than predefined variables [104]. They include interpretations of compassion as either a divine presence or a secular affirmation, revealing cultural and ontological differences. Participants may show openness or resistance, shaped by trauma or self-worth, illustrating how psychological histories influence receptivity. Experiences vary depending on whether compassion is present or absent during experiences of vulnerability, such as feeling supported (“being held in care”) or neglected (“left alone in pain”). In addition, the relational context—professional, familial, or spiritual—influences the tone and depth of compassionate encounters. Together, these contrasts deepen the understanding of compassion as a culturally embedded and ethically important phenomenon (Table 4).

**Table 4:** How to extract and code comparators in qualitative research

Study ID	Comparator type	Thematic contrast	Participant quote	Interpretation
Q01	Secular vs. religious	“It felt like divine mercy” vs. “It grounded me”	“God sent her to help me”	Religious framing deepens meaning
Q02	Acceptance vs. fear	“I couldn’t accept kindness” vs. “It saved me”	“I flinched when they hugged me”	Fear linked to trauma history
Q03	Presence vs. absence	“They were there for me” vs. “No one noticed”	“I was invisible”	Absence of compassion worsens distress

### Outcomes

The planned systematic review will explore the psychological effects of receiving compassion, focusing on primary outcomes such as emotional resilience, mental health (including depression and anxiety), and subjective well-being (including life satisfaction, emotional balance, and meaning) [105]. Secondary factors—such as self-compassion, perceived social support, and emotion regulation—will be examined as potential mediators or moderators of the relationships among these variables. Both secular and religious interpretive frameworks (e.g., ethical humanism, divine grace, and religious obligation) are evaluated for their influence on psychological outcomes [106]. Validated instruments aligned with the PRISMA 2020 guidelines will be used to assess emotion regulation.

Specifically, emotion regulation will be extracted in studies using the Emotion Regulation Questionnaire [104] and the Brief COPE Inventory [105]; self-related constructs will be extracted by the Self-Compassion Scale (SCS) [106] and the Rosenberg Self-Esteem Scale (RSES) [107]; prosociality will be obtained from studies using the Interpersonal Reactivity Index (IRI) [108], the Altruism Scale (Self-Report Altruism Scale) [109], and the Kindness Scale [110]; relationship quality will be obtained from studies using the Relational Needs Satisfaction Scale (RNSS) [111] and the Social Connectedness Scale (SCS) [112]; and resilience will be assessed using the Connor-Davidson Resilience Scale (CD-RISC) [112, 113].

Quantitative analysis will focus on studies that employ standardized tools with high internal consistency and will incorporate data such as the means, standard deviations, effect sizes (Cohen’s *d* [114], Hedges’ *g* [115]), and *p* values [116, 117]. Comparative analysis will investigate worldviews, receptivity, and exposure to compassion. Qualitative data will be analyzed using meta-ethnography to extract theories linked to the themes of compassion.

### Settings

The planned systematic review defines settings as relational or physical environments shaped by shared norms and roles, covering institutions (e.g., workplaces, schools, and religious groups) and informal encounters (e.g., public acts of kindness). Settings in which compassion is expressed will be categorized into the following domains: community-based, organizational (including clinical and hospitals), faith-based, therapeutic, and family/informal environments. Quantitative studies will extract data on variables such as the setting type, participant structure, exposure duration, delivery mode, and relational roles. In this review, we will employ meta-ethnography as the primary analytic approach to generate higher-order theoretical insights. Meta-ethnography enables the synthesis of qualitative findings across diverse study designs and allows us to extract qualitative narratives not only from qualitative research but also from the qualitative components of quantitative and mixed-method studies. These extracted narratives typically inform the outcomes domain of systematic reviews and will be used here to build interpretive explanations rather than aggregate effect sizes. Our aim is to translate concepts across studies, identify shared meanings, and construct overarching theories of receiving compassion from others. Through this interpretive synthesis, we seek to illuminate how compassion is experienced, what mechanisms shape its reception, and how these processes contribute to psychological, relational, and prosocial outcomes. 

### Information sources

We will use the following databases: CENTRAL—Cochrane Central Register of Controlled Trials, CINAHL (EBSCO), CLIB—The Cochrane Library, Embase (OVID), MEDLINE (OVID), PsycInfo (OVID), PubMed, Web of Science (Science Citation Index, Social Science Citation Index), Scopus, and Google Scholar. PubMed and MEDLINE provide indexed access to peer-reviewed health studies, offering precise MeSH-term searches that increase methodological rigor and capture key outcomes, such as psychological distress and emotional regulation [116–119]. PsycINFO offers comprehensive coverage of psychology and behavioral sciences through more than 2400 journals, supporting accurate conceptual retrieval via structured thesauri [120]. The Web of Science ensures robust citation tracking and interdisciplinary analysis, especially for healthcare policy and ethics [118, 121]. Despite concerns about reproducibility, Google Scholar supports the tracing of recent citations [119, 122]. Scopus offers a broad interdisciplinary reach and curated citation analytics [123–125], whereas CINAHL enhances nursing and allied health literature with refined search filters and controlled vocabulary [126]. Collectively, these databases form a solid foundation for examining the psychological and relational impact of compassion across various settings (Table 4) [127–129].

#### Search strategy

The planned systematic review will use a structured search strategy with multilayered eligibility criteria to ensure methodological rigor and conceptual clarity. It will include peer-reviewed, English-language studies. Although limiting the search to English only may introduce potential language bias, empirical analyses show mixed effects on the review conclusions [130–132].

A specific time restriction will not be applied to the search period for this systematic review. Imposing arbitrary date limits can reduce the comprehensiveness of the evidence base and risk excluding earlier studies that contain foundational theoretical developments or important empirical insights [76, 133]. Restricting the timeframe may also introduce selection bias by omitting relevant research simply because it predates a chosen cut-off [133, 134]. Best-practice guidance for systematic reviews emphasizes that search strategies should be as inclusive as possible to maximize transparency, reproducibility, and the completeness of evidence capture [131]. Furthermore, methodological evaluations have shown that limiting searches by date can lead to inconsistent study identification and may compromise the validity of the synthesis [135, 136]. For these reasons, expanding the search period and avoiding arbitrary temporal restrictions will ensure that all potentially relevant studies are considered, regardless of publication date.

#### Exclusion criteria

Gray literature—including dissertations, theses, conference abstracts, and unpublished reports—will be excluded from this review. This decision is based on the need to ensure methodological transparency, peer-reviewed quality assurance, and compatibility with formal risk of bias tools such as ROBINS-E, JBI, and MMAT. Peer-reviewed publications are more likely to provide complete methodological detail, validated outcome measures, and standardized reporting formats necessary for rigorous synthesis and appraisal [137]. On the other hand, gray literature may introduce interpretive ambiguity and biases due to inconsistent quality, limited peer scrutiny, and incomplete data presentation [138]. Gray literature sources often vary widely in credibility and lack formal peer-review processes, making them difficult to appraise reliably [137, 139, 140]. Their methodological reporting is frequently inconsistent or incomplete, which can compromise the ability to assess risk of bias and study validity [141]. In addition, gray literature is not systematically indexed and can be difficult to retrieve comprehensively, increasing the risk of selective capture and retrieval bias [138, 142]. Although some analyses suggest that excluding gray literature may influence effect estimates in intervention meta-analyses, the priority in this review is to maintain methodological rigor and ensure that all included studies have undergone established academic scrutiny [142].

#### Language restriction

Although compassion is a culturally embedded construct with diverse expressions across religious and secular traditions, this review restricts inclusion to English-language studies. This decision is based on pragmatic and methodological considerations. First, the review team lacks the multilingual capacity required for accurate screening, appraisal, and synthesis of non-English texts, which could introduce translation bias or misinterpretation of culturally nuanced findings [143, 144]. Second, the majority of indexed empirical literature on compassion—particularly in psychology, psychiatry, and public health—is published in English, ensuring access to a substantial and methodologically diverse evidence base [145, 146]. Third, while the exclusion of non-English studies may limit cultural generalizability, the review explicitly addresses interpretive frameworks and cultural variation within English-language contexts, including studies from South Asia, Africa, and diaspora populations [147, 148]. Future reviews may expand linguistic scope through collaborative multilingual teams to better capture global perspectives on receiving compassion.

Citation tracking, both backward and forward, will supplement database searches to increase sensitivity and address gaps in terminology and indexing [149]. Expert consultation will help refine the inclusion criteria and identify the literature where compassion intersects with psychology, philosophy, and ethics [147]. To be considered, studies must focus on adults (≥ 18 years) [150] and meet peer-review standards to ensure PRISMA-aligned reproducibility [70]. The selection process will be fully documented in a PRISMA flowchart, including manual and expert-identified sources, with clear reasons for exclusions. This iterative process will promote a robust synthesis of the psychological and relational impact of compassion across diverse settings.

### Study records

The review will follow a structured data management workflow to ensure transparency and rigor. Citations will be organized and deduplicated using EndNote 21 [151] and then screened independently and in double-anonymized mode in Rayyan to reduce bias [152]. Data extraction will be conducted using a standardized template in Excel or 133 of fields downloaded from online EndNote, capturing the study design, population characteristics, and exposure to compassion, outcomes, and contextual factors [153]. The selection decisions along with the reasons for exclusion will be documented and visualized with a PRISMA 2020 flow diagram [70]. Supporting materials, including search logs and extraction tables, will be securely stored on OneDrive for Business or SharePoint with access controls and backups [154]. To improve accessibility and reuse, files such as coded datasets and screening logs will be archived on the Open Science Framework following FAIR principles: Findable, Accessible, Interoperable, and Reusable [155].

### Data selection and extraction

To ensure transparent and thorough data collection, this review will utilize the University of Nottingham V2 Data Extraction Form developed by Santy [156] and Landis and Koch [157]. The form supports consistent methodological extraction across all eligible studies, guiding the documentation of bibliographic details, reviewer roles, eligibility decisions with rationales, and key methodological features (e.g., study aims, design, recruitment, ethics, and duration). It captures detailed participant demographics, intervention or exposure characteristics—including delivery, duration, fidelity, and comparators—and systematically records outcome measures such as definitions, instruments, psychometrics, timing, and quantitative results. It also logs missing data and reanalysis decisions. The risk of bias will be assessed across various domains, including randomization, blinding, attrition, and selective reporting, with ratings and accompanying textual justification. Additional fields include funding sources, conflicts of interest, correspondence, and interpretive notes. The form facilitates both tabular and narrative synthesis and is adaptable across various study designs, supporting reproducibility and integrity even in the case of methodological variability (Table 5).

**Table 5:** The V2 data extraction form (with hypothetical results)

Component	Planned implementation
Study identification and eligibility assessment	+ + +
Study design and methodological details	+ + +
Participant characteristics	+ + +
Intervention/exposure and comparator	+ + +
Outcome measures and follow-up timepoints	+ + +
Quantitative and qualitative results extraction	+ + +
Risk of bias assessment	+ + +
Use of validated appraisal domains	+ + −
Reporting of funding/conflicts of interest	+ + +
Space for additional notes or author correspondence	+ + +
Data extraction for synthesis-specific variables	+ − −

Legend: will be fully integrated: + + +; will be partially integrated: + + −; will require adaptation or will not be included: + − −

This review will follow a structured, multiphase approach for study selection and data extraction, ensuring transparency and consistency. All the retrieved references will be imported into EndNote 21 for deduplication and bibliographic organization and then transferred to Rayyan for blinded, independent screening of 10% of the titles and abstracts by two reviewers to establish calibration. Once alignment is achieved, a single reviewer will proceed with the remaining screenings, applying the same method to full-text assessments, with disagreements resolved through consensus or adjudication by a third reviewer [158]. Interrater reliability will be measured using Cohen’s kappa statistic, which adjusts for chance agreement and is particularly useful for qualitative synthesis and thematic coding [159, 160]. Interpretation will follow Landis and Koch’s thresholds, considering that values less than 0.60 may be insufficient in health research contexts [157]. Calculating kappa across key domains will strengthen the reliability of reviewer judgments and support thematic validity through stakeholder consultation (Table 6).

**Table 6:** Interrater reliability matrix for concordance between two reviewers regarding the inclusion criteria

	Rater 1: paper meets the criteria	Rater 1: paper does not meet the criteria
Rater 2: paper meets the criteria	[n]	[n]
Rater 2: paper does not meet the criteria	[n]	[n]

The review will include expert consultation to identify eligible studies beyond traditional databases, thus enhancing the coverage of complex concepts [72]. Data extraction will follow a predefined and tested data abstraction table (DAT) created in Excel [161]. Specifically, study metadata (author, year, country, design, setting, sample size, demographics, eligibility criteria, sample size, outcomes, journal, and doi) will be recorded, and detailed documentation of compassion exposure (nature, source, frequency, framing), comparators, psychological outcomes (e.g., resilience, distress), and key findings will be included [162, 163]. Additional fields will document the relational context, recruitment methods, cultural framing, measurement tools, analytic strategies, limitations, and bias risks. The notes will include duplicates, follow-ups, reviewer comments, and unresolved questions. All entries will use a dd/mm/yyyy format and be verified by a second reviewer. Any unclear or missing data will be addressed by contacting the authors [163], and the exclusion decisions will be presented in a PRISMA 2020 flow diagram [70]. The finalized DAT will support both qualitative and quantitative analyses.

### Eligible studies

Eligible studies must be empirical (i.e., quantitative, qualitative, or mixed methods) and explicitly examine the experience of receiving compassion as an interpersonal construct linked to emotional or psychological outcomes. Quantitative designs include RCTs, quasi-experiments, and observational studies (e.g., cohort, case–control, cross-sectional) using validated measures such as the Compassionate Engagement and Action Scale—Receiving subscale to assess outcomes such as resilience or emotional regulation. Qualitative approaches (e.g., phenomenology, grounded theory, ethnography, narrative inquiry, case studies) must explore the lived experiences of receiving compassion through interviews, diaries, or observational methods. Mixed-methods studies are eligible if both data strands are integrated and if compassion-as-received is a core construct. Only studies involving human participants in naturalistic, clinical, organizational, educational, community, or religious/spiritual contexts will be included. Compassion must be explicitly measured via validated tools, behavioral observation, or participant narratives with clear relevance to well-being. Studies that lack empirical data, focus solely on providing compassion or adjacent constructs (e.g., empathy and social support), involve animals, consider hypothetical scenarios, or treat compassion as a peripheral theme will be excluded.

### Data items

This review will extract variables using a modified PI/ECOS framework enriched with contextual and methodological dimensions essential for understanding received compassion and its psychological effects. Study identification will include a record of the study metadata (author, year, country, journal, DOI, funding, conflicts of interest) to ensure transparency. The population data will detail the demographics, eligibility criteria, and recruitment strategies. The settings will be categorized (clinical, organizational, educational, community, spiritual/religious), and the exposure variables will cover operationalization, source, delivery mode, frequency, duration, and framing. Validated instruments, such as the Compassionate Engagement and Action Scale—Receiving subscale, will be used for quantitative studies. In contrast, qualitative and mixed-methods studies will prioritize experiential definitions. Comparator information will include care conditions, exposure types, and philosophical framing. Outcomes related to receiving compassion will be assessed using a suite of validated standardized measures that capture both psychosocial well-being and the multidimensional experience of compassion from others. These instruments will be selected because they are widely used in empirical studies examining compassion, affiliative relating, and interpersonal warmth, and are therefore likely to be identified in the published literature included in this review. General well-being will be assessed using the WHOQOL-BREF, a widely used instrument that evaluates physical, psychological, social, and environmental quality of life [164]. Perceived compassion directed toward the individual is usually measured using Asano et al.’s Compassion from Others Scale, which quantifies the extent to which individuals feel cared for, supported, and emotionally understood by others [165]. Defensive or avoidant responses to receiving compassion are usually assessed using Gilbert, McEwan et al.’s Fear of Compassion from Others Scale, which evaluates difficulties in tolerating or trusting compassion offered by others [3]. Openness to receiving compassion is usually measured using Gilbert, Catarino et al.’s Receiving/Accepting Compassion From Others Scale, which assesses an individual’s capacity to allow, internalize, and benefit from compassion offered by others [96]. Additional relational and affective dimensions of received compassion are usually captured using Sprecher and Fehr’s Compassionate Love Scale, which measures altruistic, other-oriented compassion within interpersonal relationships [166], and Gilbert, McEwan, Mitra et al.’s Social Safeness and Pleasure Scale, which assesses perceived warmth, acceptance, and affiliative connection from others—key components of feeling safe within compassionate social environments [167]. Together, these instruments will provide a comprehensive assessment of the psychosocial impact of receiving compassion across emotional, relational, and well-being domains and will reflect the measures most used in compassion-focused research.

Qualitative outcomes in compassion research will typically appear as narrative accounts describing how individuals, in community, hospitals (either as health carers of patients), and other organizations, experience and interpret compassion received from others. These findings will often be derived from interviews, focus groups, or open-ended survey responses and will highlight themes such as feeling cared for, emotionally supported, safe, or connected. Studies will also report barriers to receiving compassion—such as shame, mistrust, or fear—and the influence of cultural, interpersonal, and secular or religious meaning-making frameworks. These qualitative insights will complement quantitative measures by illuminating the subjective and contextual dimensions of receiving compassion [168, 169].

Methodological variables (design, sampling, duration, analytic techniques, blinding, and integration) and contextual framings (e.g., moral, religious, and ethical) will be used to support cross-study comparisons. Funding and conflict disclosures will be documented, and extraction will follow planned simplifications, including the merging of outcomes and prioritizing key timepoints. Thematically coded qualitative data will be consolidated conceptually, with missing information verified through contact with the authors. All entries will be reviewed using a structured data abstraction table.

## Outcomes and prioritization

### Outcomes of interest

This review will focus on mental health, psychological well-being, and prosocial behaviors as its primary outcomes, defined as emotional and cognitive functioning following compassion from others. The specific measures include depression, anxiety, psychological distress, subjective well-being, life satisfaction, social behaviors, and psychological flourishing, which will be assessed through validated tools across baseline, follow-up, or cross-sectional data. Secondary outcomes will include emotional regulation, coping skills, and self-perception (e.g., self-worth, self-compassion, self-esteem), as well as prosocial behaviors such as empathy, kindness, and altruism, which will be measured through self-reports or behavioral observations. Indicators of long-term resilience and psychological adaptation after adversity will also be documented, with outcome timing noted to allow analysis of short- versus long-term effects.

## Risk of bias in individual studies

To ensure methodological rigor and transparency, the planned review will assess the risk of bias using design-specific critical appraisal tools validated for systematic reviews, relying primarily on the Cochrane Risk of Bias Tool for Non-Randomized Studies—of Exposures (ROBINS-E) [72], owing to the likely absence of randomized controlled trials (RCTs). ROBINS-E examines bias across seven domains—confounding, exposure measurement, participant selection, postexposure interventions, missing data, outcome measurement, and result selection; and each study will be systematically evaluated to assess overall bias [72]. The results will be showcased through a semaphore-style visual summary to enhance interpretive clarity and facilitate transparent cross-design comparisons according to PRISMA guidelines (Table 7).

**Table 7:** Semaphore-style chart for ROBINS-E (hypothetical studies)

Study ID	Bias due to confounding	Bias in selection of participants	Bias in exposure classification	Bias due to missing data	Bias in measurement of outcomes	Bias in selection of reported results	Overall ROB
Study A	M	M	L	L	M	L	M
Study B	S	M	L	L	S	L	S
Study C	L	L	M	L	L	M	M
Study D	C	M	S	L	M	S	C
Study E	L	L	L	M	L	L	L

Legend: *L *low, *M *moderate, *S *serious, *C *critical

For quantitative studies employing pre–post or quasi-experimental designs without randomization and for qualitative studies, this review will utilize the Joanna Briggs Institute (JBI) Critical Appraisal Checklist, which comprises nine structured items that assess design integrity: temporal clarity between exposure and outcome, group comparability, consistency of care aside from intervention, presence of a control group, measurement before and after the intervention, follow-up completeness, reliability of measurement, and statistical appropriateness [170, 171] (Table 8).

**Table 8:** Semaphore-style chart for JBI critical appraisal checklist for quasi-experimental studies (hypothetical studies)

Study	Clarity of cause-and-effect relationship	Similarity of participants	Consistency of treatment/care (excl. intervention)	Presence of a control group	Pre/post outcome measurements	Completeness of follow-up	Outcome measurement consistency	Outcome measurement reliability	Appropriate statistical analysis
Study A	FA	Fa	PA	PA	FA	PA	FA	FA	FA
Study B	FA	PA	FA	NA	PA	FA	FA	FA	FA
Study C	FA	FA	FA	FA	FA	PA	FA	PA	FA
Study D	PA	NA	PA	FA	FA	NA	PA	FA	PA

Legend: *FA *fully addressed, *PA *partially addressed, *NA *not addressed

The Joanna Briggs Institute (JBI) also provides one of the most structured and explicit frameworks for assessing bias in qualitative studies. Its Critical Appraisal Checklist for Qualitative Research evaluates methodological congruence, researcher reflexivity, representation of participants’ voices, and the transparency of interpretive processes. JBI explicitly examines how researchers’ assumptions, positionality, and interactions with participants may introduce bias, and whether these influences are acknowledged and managed. It also assesses the alignment between philosophical underpinnings, methodology, data collection, and analysis—key indicators of internal validity in qualitative inquiry. By requiring clear documentation of analytic decisions and interpretive pathways, JBI strengthens the dependability, credibility, and confirmability of qualitative evidence, making it a rigorous tool for evaluating trustworthiness and reducing interpretive bias [172].

Each item will be rated “Yes,” “No,” “Unclear,” or “Not applicable,” with the aggregate judgment informing study inclusion and synthesis weighting, thereby enhancing transparency and reproducibility, as shown in Table 8.

To complement the Mixed Methods Appraisal Tool (MMAT), the Joanna Briggs Institute (JBI) Critical Appraisal Checklist for Qualitative Research will be used to evaluate the methodological quality of standalone qualitative studies and qualitative components within mixed-methods designs [173]. This ten-item tool assesses the alignment between philosophical orientation and methodological choices, including data collection, analysis, and interpretation, as well as reflexivity, ethics, and the credibility and transferability of findings [174]. Each domain will be rated independently by two reviewers, with disagreements resolved through discussion or third-party input. The results will be summarized using a semaphore-style chart to visually display the methodological strengths and limitations across JBI domains, as shown in Table 9.

**Table 9:** Semaphore-style chart for JBI for qualitative studies (hypothetical studies)

Study ID	Congruity between philosophy and methodology	Congruity between methodology and research question	Congruity in data collection methods	Representation of the researcher’s position	Influence of the researcher on research	Participant representation	Ethical considerations	Rigor of data analysis	Link between data & interpretation	Conclusions flow from analysis	Overall ROB
Study A	LR	LR	SC	LR	LR	LR	LR	SC	LR	LR	SC
Study B	SC	SC	HR	SC	HR	SC	HR	HR	SC	HR	HR
Study C	LR	LR	LR	SC	SC	LR	LR	LR	LR	LR	LR
Study D	HR	HR	SC	NI	HR	SC	NI	HR	SC	HR	HR
Study E	LR	LR	LR	LR	SC	LR	SC	LR	LR	LR	LR

Legend: *LR *low risk, *SC *some concern, *HR *high risk, *NI *no information

Mixed-methods studies will be evaluated using the Mixed Methods Appraisal Tool (MMAT) [173]. MMAT can be used for reviews using various methodologies and assesses five key criteria: the rationale for the mixed-methods design, the effectiveness of integration, the interpretation of the combined results, the handling of inconsistencies, and the methodological quality within each strand [174]. Two reviewers will independently evaluate each study, resolving any disagreements through discussion or by seeking input from a third party [175]. The results will be displayed using a semaphore-style chart to highlight strengths and limitations across MMAT domains (Table 10).

**Table 10:** Semaphore-style chart for MMAT appraisal of mixed-methods studies (hypothetical studies)

Study	Rationale for using mixed methods	Integration of components	Interpretation of findings	Addressing divergences	Methodological quality of each component
Study A	FA	FA	PA	PA	FA
Study B	FA	FA	FA	NA	FA
Study C	PA	FA	FA	PA	PA
Study D	FA	FA	PA	PA	FA

Legend: *FA *fully addressed, *PA *partially addressed or unclear, *NA *not addressed

Each included study will be independently critically appraised by two reviewers, with any disagreements resolved through discussion or third-party adjudication. The results of these risk of bias assessments will be summarized both narratively and in tabular form to enhance methodological transparency and facilitate detailed interpretation across different study designs.

### Integration of bias assessment

To ensure coherence across diverse study designs, the review will apply a standardized mapping of bias domains. This means that although different tools are used—such as RoB 2.0 for randomized trials, ROBINS-I for non-randomized studies, JBI for qualitative research, MMAT for mixed-methods, and GRADE-Pro for outcome-level certainty—their judgments will be interpreted through a common lens. For example, domains like selection bias, performance bias, detection bias, attrition bias, and reporting bias will be aligned conceptually across tools, allowing for consistent comparison and synthesis. In practice, this means that a study judged to have “serious risk of bias due to confounding” in ROBINS-I will be interpreted alongside a study with “some concerns in randomization” under RoB 2.0, both contributing to a broader understanding of selection bias across the evidence base. This harmonization enables the review to compare methodological quality across heterogeneous designs without distorting the meaning of each tool’s criteria [176].

The results of these assessments will be synthesized narratively. The review will describe patterns of bias across studies, noting which domains were most frequently compromised and how these limitations affect confidence in the findings. For example, the synthesis may report that most randomized trials showed low risk of bias, while non-randomized studies were more vulnerable to confounding and misclassification. Qualitative studies may be described as methodologically congruent but occasionally lacking clarity in sampling procedures. These narrative judgments will inform the weighting of evidence in the final synthesis. Studies with high risk of bias will be down-weighted in GRADE certainty ratings and explored through sensitivity analyses. The narrative will explicitly link methodological limitations to the interpretation of results, ensuring that conclusions are grounded in the quality of the underlying evidence (Table 11) [177].

**Table 11:** Integrated appraisal domains across MMAT, JBI, and ROBINS-E

Unified domain	MMAT (Mixed Methods Appraisal Tool)	JBI (Joanna Briggs Institute)	ROBINS-E (Risk of Bias in Non-randomized Studies—Exposure)
1. Research aim and design	Appropriateness of study design to research question	Congruity between research methodology and objectives	Bias due to confounding; clarity of causal question
2. Sampling and recruitment	Relevance and appropriateness of sampling strategy	Appropriateness of participant recruitment and inclusion criteria	Bias in selection of participants into the study
3. Data collection	Adequacy and relevance of data collection methods	Congruity between data collection methods and research methodology	Bias in classification of exposures and measurement of variables
4. Intervention/exposure integrity	Intervention integrity (if applicable)	Intervention fidelity and contextual relevance	Bias due to deviations from intended exposures
5. Outcome measurement	Appropriateness of outcome measures	Validity and reliability of outcome measures	Bias in measurement of outcomes
6. Data analysis	Appropriateness of statistical or qualitative analysis	Congruity between data analysis and research methodology	Bias due to missing data; selective reporting
7. Reflexivity and ethics	Consideration of researcher influence and ethical issues	Ethical approval; researcher positionality and influence	Not directly assessed
8. Integration and interpretation	Coherence between qualitative and quantitative components (MM)	Interpretation of results supported by data	Bias in selection of reported results
9. Overall risk of bias	Overall appraisal per component and study type	Overall appraisal of methodological quality	Overall risk of bias judgment across domains

## Data synthesis

### Quantitative data synthesis

This systematic review will include a meta-analysis to produce statistically precise estimates of the overall effect of receiving compassion on psychological well-being by combining eligible quantitative findings [178, 179]. A random-effects model will be used to account for between-study variability and to enhance generalizability [180]. Effect sizes will be calculated primarily using Cohen’s *d*, facilitating comparisons across diverse outcome measures, with thresholds of 0.2 (small), 0.5 (medium), and ≥ 0.8 (large) [181]. Heterogeneity will be assessed with the I^2^ statistic: values < 40% suggest low variability, whereas values > 75% may require subgroup analysis or meta-regression [178]. Publication bias will be examined using Kendall’s tau test within Begg’s test for funnel plot asymmetry, which is appropriate for small or nonnormally distributed samples despite being less sensitive than Egger’s test [182]. Statistical synthesis will be performed using Comprehensive Meta-Analysis software [183], with forest plots displaying pooled estimates and 95% confidence intervals to aid clinical interpretation [184]. When applicable, Cohen’s *d* values will be used to indicate overall psychological impact, and Pearson’s *r*, which reflects the strength and direction of linear relationships, will be extracted, with thresholds of 0.10 (small), 0.30 (medium), and ≥ 0.50 (large) [185].

### Qualitative data synthesis and meta-ethnographic synthesis

Qualitative data synthesis, or the integration of all qualitative data, such as the description of the outcomes of quantitative and mixed-methods studies, will be carried out using a meta-ethnographic analysis. This method will facilitate the integration of findings from qualitative studies, as well as qualitative outcomes, within mixed-methods or quantitative research [186–189]. When the development of theoretical concepts is a key aim, meta-ethnography will be employed to enhance interpretive depth [190, 191]. Meta-ethnography supports theory building in compassion research because it synthesizes qualitative studies at the level of meaning, enabling the development of new conceptual insights. Through translation of concepts across contexts, preservation of interpretive depth, and integration of diverse relational perspectives, meta-ethnography generates higher-order theoretical models that explain how compassion is understood, enacted, and shaped by environments [155]. Meta-ethnographic analysis, which was first developed by Noblit and Hare [186, 191] and later adapted by Sattar et al. [192], is a qualitative synthesis method used in systematic reviews to generate new theoretical insights through the interpretation of findings across multiple studies. This iterative approach begins with a clearly defined research question, followed by systematic identification and a close reading of relevant studies. Researchers then explore relationships between studies by identifying recurring concepts or metaphors, which enables the translation of themes across studies, aligning terminologies and revealing both reciprocal (similar) and refutational (divergent) relationships. The final stage involves constructing a “line of argument,” an integrated theoretical narrative that transcends individual study conclusions, producing rich, practice-informed understandings that are both contextually grounded and conceptually expansive for policy and future research (Table 12) [155].

**Table 12:** The seven steps in meta-ethnographic analysis for five dummy studies (A–E) [see [155, 186, 191–197]]

Meta-ethnography step	Description of step	Application to 5 dummy compassion studies
1. Getting started	Identify an area where conceptual understanding is needed	Focus: *How compassion is understood, enacted, and constrained in healthcare settings*
2. Deciding what is relevant	Select qualitative studies meeting inclusion criteria or qualitative synthesis of quantitative studies’ outcomes	Study A: nurses’ lived experiences of compassion Study B: medical students’ barriers to compassionate care Study C: compassion in palliative care teams Study D: patient experiences of receiving compassion Study E: organizational influences on compassionate practice
3. Reading the studies	Extract first-order (participant) and second-order (author) constructs	Key constructs identified: emotional attunement, moral sensitivity, compassion fatigue, institutional pressure, relational presence, empathy-in-action
4. Determining how the studies are related	Compare concepts across studies to identify similarities and differences	Shared themes: relational attunement (A, C, D), emotional labor (A, B), systemic constraints (B, E), compassion as co-constructed (C, D)
5. Translating the studies into one another	Conduct reciprocal and refutational translation	Reciprocal: compassion requires emotional presence (A, C, D) Refutational: compassion as innate (A, D) vs. compassion as teachable (B)
6. Synthesizing translations	Develop third-order constructs (new interpretations)	Three emergent constructs: 1. Compassion as relational attunement (integration of A, C, D) 2. Compassion under pressure (B, E) 3. Compassion as a dynamic moral practice (all studies)
7. Expressing the synthesis	Present the new theory or model	Resulting model: *A multi-layered theory of compassion* linking individual emotional capacities, relational processes, and organizational conditions

Meta-ethnography, as outlined by Ring et al., has a significant impact on qualitative evidence synthesis because it moves beyond description to generate new conceptual and theoretical insights. By translating concepts across studies, it enables researchers to build higher-order explanations that clarify how and why health interventions work in real-world contexts. This interpretive depth strengthens public health decision-making, enhances the explanatory power of systematic reviews, and supports the development of models that inform policy, service design, and patient-centered care [197]. Meta-ethnography enhances validity by grounding its interpretations in systematic, transparent translation of concepts across studies, ensuring that higher-order constructs remain faithful to the meanings expressed in the primary data. Reliability is strengthened through iterative team-based reading, constant comparison, and explicit documentation of analytic decisions, which together support consistency and auditability of the synthesis. The method’s emphasis on conceptual translation rather than aggregation allows for robust theoretical development while maintaining interpretive rigor [194, 198, 199].

### Evaluating the robustness of the narrative synthesis

To evaluate the robustness of the narrative synthesis, this review will critically examine the coherence of findings across studies, the potential influence of study quality, and the reliance on particularly dominant sources while assessing contextual relevance and alignment with the primary research question. Methodological dependability will be evaluated through criteria adapted from Popay et al. [200], including the consistency of the translated findings, the impact of variability in the study quality, and the distribution of influence across studies. To enhance credibility, a face validity check will be conducted with experts in compassion science, mental health practitioners, commissioners, and other stakeholders. Their feedback will be analyzed using correlation matrices and chi-square tests to determine alignment with the synthesized conceptual framework. Reviewer agreement patterns will be summarized, as shown in Table 11 [201]. This process aims to ensure that the final synthesis reflects both methodological integrity and practical relevance across diverse contexts (Table 13).

**Table 13:** Table of agreement for the robustness of the narrative synthesis of the SR (hypothetical summary results for positive answers) (see [200])

Domains	Reviewer 1	Reviewer 2	Reviewer 3
Consistent translation of findings between studies	X	–	X
The results appear to be biased based on the quality of the contributing studies	–	X	
The studies included in the synthesis are reliable	X	X	–
The results appear to depend heavily on the credibility of the synthesis	X		
The results appear to vary depending on the context	X	–	X
The results answer the review question	X	X	X

### Meta-biases

#### Confidence in cumulative evidence

The GRADEpro GDT platform will support evidence assessment and facilitate the transparent synthesis of confidence in the findings [202, 203]. The strength of the evidence in this systematic review will be evaluated using the GRADE (Grading of Recommendations, Assessment, Development, and Evaluation) approach, implemented through the GRADEpro GDT software. This method provides a transparent and structured approach for evaluating the certainty of evidence across outcomes and summarizing findings in a manner that facilitates informed decision-making in health research and policy [204, 205].

#### GRADE domains for certainty assessment

GRADE assesses the certainty of evidence for each outcome across five main domains [206, 207]:

1. Risk of bias—evaluates the methodological limitations of the included studies (e.g., lack of blinding and incomplete outcome data).
2. Inconsistency—examines the variability in the results across studies (e.g., heterogeneity in effect sizes).
3. Indirectness—considers how applicable the evidence is to the review question (e.g., differences in population, intervention, or outcomes).
4. Imprecision—reviews the confidence intervals for the effect estimates (e.g., wide intervals or small sample sizes).
5. Publication bias—examines the likelihood of the selective publication of studies with positive results.

Each outcome starts with high certainty for randomized trials or low certainty for observational studies and can be downgraded or upgraded based on these domains. The final rating is categorized as having high, moderate, low, or very low certainty.

#### Application in GRADEpro GDT

GRADEpro GDT (https://www.gradepro.org) is the official software created by the GRADE Working Group. It helps in creating the following:

1. Summary of Findings (SoF) tables, which show the effect estimates, certainty levels, and key outcome data.
2. Evidence Profiles, which provide detailed reasons for each domain rating and promote transparent decision-making.

For example, in a review evaluating the impact of receiving compassion on psychological distress, the outcome “depression” might be rated as moderate certainty if the included studies are randomized but display some inconsistency and imprecision. The SoF table would display the combined effect size (e.g., Cohen’s *d* = − 0.45), the number of participants, and the GRADE rating, along with footnotes explaining any downgrades [164, 208–210] (Table 14).

**Table 14:** In a Cochrane-style review of compassion-based interventions, GRADEpro GDT might be used to summarize outcomes (hypothetical results)

Outcome	Effect estimate (Cohen’s *d*)	Participants	Studies	Certainty
Depression	− 0.45 (95% CI: − 0.70 to − 0.20)	1200	8	Moderate
Anxiety	− 0.30 (95% CI: − 0.55 to − 0.05)	950	6	Low
Psychological flourishing	+ 0.40 (95% CI: + 0.10 to + 0.70)	600	4	Moderate

Each rating would be justified in the Evidence Profile, citing reasons such as “downgraded for imprecision due to wide confidence intervals” or “no serious risk of bias” (Table 15).

**Table 15:** GRADE-Pro GDT summary of findings table: effects of receiving compassion on psychological outcomes (hypothetical variables)

Outcome	No. of participants (studies)	Study design	Risk of bias	Inconsistency	Indirectness	Imprecision	Publication bias	Overall certainty	Effect estimate (95% CI)	Comments
Depression	1200 (8 studies)	RCTs + observational	Not serious	Moderate	Not serious	Serious	Possible	⬤⬤⬤◯ Moderate	Cohen’s *d* = − 0.45 (− 0.70 to − 0.20)	Downgraded for imprecision due to varied measures and small subsamples
Anxiety	950 (6 studies)	RCTs + observational	Serious	Moderate	Not serious	Serious	Undetected	⬤⬤◯◯ Low	Cohen’s *d* = − 0.30 (− 0.55 to − 0.05)	Downgraded for risk of bias and imprecision
Psychological flourishing	600 (4 studies)	Observational	Not serious	Serious	Serious	Not serious	Likely	⬤⬤◯◯ Low	Cohen’s *d* = + 0.40 (+ 0.10 to + 0.70)	Downgraded for inconsistency and indirectness
Resilience	800 (5 studies)	RCTs + mixed	Not serious	Not serious	Not serious	Serious	Possible	⬤⬤⬤◯ Moderate	Cohen’s *d* = + 0.35 (+ 0.12 to + 0.58)	Downgraded for small sample sizes in subgroup studies
Emotional regulation	700 (4 studies)	Observational	Serious	Moderate	Not serious	Serious	Possible	⬤⬤◯◯ Low	Cohen’s *d* = + 0.28 (+ 0.03 to + 0.53)	Downgraded due to variability and measurement inconsistency

### Integrated theoretical model of compassion reception: a mixed-methods synthesis

This systematic review will adopt a mixed-methods approach to develop an integrated theoretical model of compassion reception across clinical, secular, and spiritual contexts. *In the quantitative strand*, meta-analyses will be conducted across predefined outcome domains—such as depression, burnout, emotional regulation, and social safeness—pooling effect sizes and assessing heterogeneity to identify patterns of association between compassion from others and psychological or relational outcomes. *In the qualitative strand*, meta-ethnography will be used to extract experiential mechanisms from eligible studies, with anticipated theories including relational safety, emotional openness, moral purpose, and resilience through connection. *The analysis will initially proceed in parallel*, with each strand approached independently to preserve methodological integrity and epistemological distinctiveness. *Convergence will occur in the integration phase*, where findings from each strand will be aligned within a shared conceptual framework using joint display techniques—such as annotated forest plots, quadrant matrices, and conceptual graphs—to visually and interpretively merge statistical outcomes with thematic insights. *Selective data transformation* may also be employed to enhance coherence, for example, by mapping qualitative constructs onto quantitative outcome domains or interpreting pooled effect sizes as experiential categories. The *final narrative*, emerging from merging the meta-ethnographic outcomes from the studies, will support the generation of both a middle-range and a grand theory of received compassion through meta-integration. A *middle-range theory* will offer a context-sensitive, testable framework that explains how compassion from others operates within specific populations and settings—such as clinical care, education, or spiritual practice—while a *grand theory* will aim to articulate compassion reception as a universal, multi-level construct encompassing affective, relational, and ethical dimensions. Together, these theoretical outputs will be adaptable for clinical governance, pedagogical translation, and policy development, advancing a reproducible and conceptually rich understanding of compassion’s role in human flourishing.

### Domain-based harmonization of risk-of-bias judgements in multi-design reviews

To ensure methodological coherence while avoiding false equivalence across heterogeneous study designs, risk-of-bias (RoB) assessments will be harmonized using a domain-based, design-sensitive approach. Consistent with guidance from the *Cochrane Handbook for Systematic Reviews of Interventions*, global RoB scores will not be calculated, as such scores can obscure design-specific vulnerabilities and imply comparability between fundamentally different forms of bias (Higgins et al. [76]). Instead, each study will be appraised using the tool appropriate to its design—such as RoB2 or ROBINS-I for quantitative studies and the Joanna Briggs Institute checklist for qualitative and mixed-methods research—recognizing that quantitative tools assess threats such as randomization, confounding, and outcome measurement, whereas qualitative tools evaluate congruity, reflexivity, and interpretive coherence [172]. To enable structured synthesis, tool-specific domains will then be mapped onto higher-order conceptual categories (e.g., selection bias, measurement bias, reporting bias, interpretive bias) without assuming equivalence between them.

This approach aligns with methodological reviews demonstrating that reporting and methodological biases manifest differently across designs and therefore require design-appropriate interpretation rather than numerical aggregation (Page, Higgins, Clayton, Sterne, Hróbjartsson, and Savović). Final synthesis retained the original tool-specific judgments and used narrative integration to ensure transparency and avoid misleading cross-study comparisons [211].

## Discussion

Our planned systematic review aims to synthesize evidence on the psychological effects of receiving compassion from others, focusing on how these experiences are interpreted through secular and religious frameworks. It is expected that the review will find consistent links between receiving compassion and observed improvements in psychological well-being, including reduced distress, increased resilience, and improved emotional functioning. These outcomes are likely to align with existing research suggesting that compassion—whether expressed through interpersonal care, community support, or spiritual practices—can serve as a buffer against psychological adversity. The review will also examine how interpretive frameworks influence the experience and effects of compassion. Religious views may frame compassion within doctrines of grace, forgiveness, or divine presence, whereas secular views may emphasize empathy, dignity, and shared humanity. Both perspectives are expected to produce positive psychological outcomes, potentially through different mechanisms such as meaning-making or social connectedness. When enough data are available, pooled estimates of effect size (e.g., Cohen’s *d*) will be calculated to measure the overall impact of receiving compassion on psychological well-being. Forest plots with 95% confidence intervals will be used to visually summarize the effects across studies.

This review will explore how receiving compassion—interpreted through secular or religious lenses—affects psychological well-being, including potential reductions in distress and increases in resilience. By synthesizing evidence across diverse settings and study designs, the review aims to develop culturally sensitive and spiritually aware models of compassionate interaction.

The expected findings have direct translational relevance across multiple domains:

- Clinical practice: Insights into how individuals receive and interpret compassion can inform therapeutic formulations, especially in contexts involving trauma, shame, or relational fear. Mental health professionals may use the findings to enhance compassion-focused interventions, improve therapeutic alliances, and foster emotionally safe environments in multidisciplinary care.
- Educational settings: The review will support the integration of compassion literacy into health and social care curricula, equipping future practitioners with evidence-based understanding of relational care. It may also inform professional development programs in emotional intelligence, reflective practice, and trauma-informed pedagogy, particularly in high-stress learning environments.
- Community and public health contexts: Findings may guide the design of compassion-based initiatives in secular and faith-based communities, promoting resilience and social connectedness. Public health campaigns and organizational policies can draw on the evidence to cultivate compassionate cultures, reduce stigma, and support recovery in post-crisis settings.

By clarifying the psychological effects of receiving compassion and the interpretive frameworks that shape its impact, this review contributes to interdisciplinary models of care that are ethically grounded, contextually responsive, and practically applicable across clinical, educational, and community domains.

### Limitations

In accordance with the 2020 PRISMA guidelines, several limitations are anticipated. First, restricting inclusion to English-language publications may introduce language bias. Second, heterogeneity in compassion studies, outcome measures, and study populations may limit the ability to conduct meta-analyses or draw generalizable conclusions. Third, although interrater reliability will be established during screening and data extraction, using a single reviewer for most records may introduce selection bias. Finally, the GRADE approach will be used to assess the certainty of the evidence, which might be rated as low or moderate because of methodological limitations in primary studies. Despite these limitations, the review aims to provide a structured and transparent synthesis of evidence on the psychological effects of receiving compassion, offering insights into how secular and religious interpretations may influence these outcomes. The findings will guide future research and practices in clinical, community, and spiritual care settings.

## Data Availability

The protocol is available on the PROSPERO website.

## Appendix

### PRISMA-P 2015 Checklist

1. This checklist has been adapted for use with systematic review protocol submissions to BioMed Central journals, based on Table 3 in Moher D et al. (2015): Preferred reporting items for systematic review and meta-analysis protocols (PRISMA-P) 2015 statement. *Systematic Reviews* 2015 4:1
2. An Editorial from the Editors-in-Chief of *Systematic Reviews* details why this checklist was adapted - Moher D, Stewart L & Shekelle P: Implementing PRISMA-P: recommendations for prospective authors. *Systematic Reviews* 2016 5:15

**Table:**

Section/topic	#	Checklist item	Information reported		Line number(s)
			Yes	No	
ADMINISTRATIVE INFORMATION					
Title					
Identification	1a	Identify the report as a protocol of a systematic review	☒	☐	
Update	1b	If the protocol is for an update of a previous systematic review, identify as such	☐	☒	
Registration	2	If registered, provide the name of the registry (e.g., PROSPERO) and registration number in the Abstract	☒	☐	
Authors					
Contact	3a	Provide name, institutional affiliation, and e-mail address of all protocol authors; provide physical mailing address of corresponding author	☒	☐	
Contributions	3b	Describe contributions of protocol authors and identify the guarantor of the review	☒	☐	
Amendments	4	If the protocol represents an amendment of a previously completed or published protocol, identify as such and list changes; otherwise, state plan for documenting important protocol amendments	☒	☐	
Support					
Sources	5a	Indicate sources of financial or other support for the review	☒	☐	
Sponsor	5b	Provide name for the review funder and/or sponsor	☐	☒	
Role of sponsor/funder	5c	Describe roles of funder(s), sponsor(s), and/or institution(s), if any, in developing the protocol	☐	☒	
INTRODUCTION					
Rationale	6	Describe the rationale for the review in the context of what is already known	☒	☐	
Objectives	7	Provide an explicit statement of the question(s) the review will address with reference to participants, interventions, comparators, and outcomes (PICO)	☒	☐	
METHODS					
Eligibility criteria	8	Specify the study characteristics (e.g., PICO, study design, setting, time frame) and report characteristics (e.g., years considered, language, publication status) to be used as criteria for eligibility for the review	☒	☐	
Information sources	9	Describe all intended information sources (e.g., electronic databases, contact with study authors, trial registers, or other grey literature sources) with planned dates of coverage	☒	☐	
Search strategy	10	Present draft of search strategy to be used for at least one electronic database, including planned limits, such that it could be repeated	☒	☐	
*STUDY RECORDS*					
Data management	11a	Describe the mechanism(s) that will be used to manage records and data throughout the review	☒	☐	
Selection process	11b	State the process that will be used for selecting studies (e.g., two independent reviewers) through each phase of the review (i.e., screening, eligibility, and inclusion in meta-analysis)	☒	☐	
Data collection process	11c	Describe planned method of extracting data from reports (e.g., piloting forms, done independently, in duplicate), any processes for obtaining and confirming data from investigators	☒	☐	
Data items	12	List and define all variables for which data will be sought (e.g., PICO items, funding sources), any pre-planned data assumptions and simplifications	☒	☐	
Outcomes and prioritization	13	List and define all outcomes for which data will be sought, including prioritization of main and additional outcomes, with rationale	☒	☐	
Risk of bias in individual studies	14	Describe anticipated methods for assessing risk of bias of individual studies, including whether this will be done at the outcome or study level, or both; state how this information will be used in data synthesis	☒	☐	
*DATA*					
Synthesis	15a	Describe criteria under which study data will be quantitatively synthesized	☒	☐	
	15b	If data are appropriate for quantitative synthesis, describe planned summary measures, methods of handling data, and methods of combining data from studies, including any planned exploration of consistency (e.g., *I* 2, Kendall’s tau)	☒	☐	
	15c	Describe any proposed additional analyzes (e.g., sensitivity or subgroup analyzes, meta-regression)	☒	☒	
	15d	If quantitative synthesis is not appropriate, describe the type of summary planned	☒	☐	
Meta-bias(es)	16	Specify any planned assessment of meta-bias(es) (e.g., publication bias across studies, selective reporting within studies)	☒	☐	
Confidence in cumulative evidence	17	Describe how the strength of the body of evidence will be assessed (e.g., GRADE)	☒	☐	

## Abbreviations

AMSTAR: A Measurement Tool to Assess Systematic Reviews
CONSORT: Consolidated Standards of Reporting Trials
Cochrane RoB 2: Cochrane Risk of Bias Tool (for randomized trials)
GRADE: Grading of Recommendations, Assessment, Development, and Evaluation
GRADE-Pro: GRADE Profiler software
JBI-QARI: Joanna Briggs Institute Qualitative Assessment and Review Instrument
MMAT: Mixed Methods Appraisal Tool
PRISMA: Preferred Reporting Items for Systematic Reviews and Meta-Analyses
PRISMA-P: PRISMA for Protocols
PROSPERO: International Prospective Register of Systematic Reviews
ROBINS: Risk of Bias in Systematic Reviews
